# Seasonal changes in activity of hypothalamic thyroid hormone system in different winter phenotypes of Djungarian hamster (*Phodopus sungorus*)

**DOI:** 10.1371/journal.pone.0309591

**Published:** 2024-10-25

**Authors:** Anna S. Przybylska-Piech, Victoria Diedrich, Annika Herwig

**Affiliations:** 1 Department of Vertebrate Zoology and Ecology, Nicolaus Copernicus University in Toruń, Toruń, Poland; 2 Institute of Neurobiology, Ulm University, Ulm, Germany; Indian Institute of Technology Indore, INDIA

## Abstract

Although the Djungarian hamster (*Phodopus sungorus*) is a seasonality model, it presents substantial variability in winter acclimation. In response to short photoperiod, some individuals express a suite of winter traits such as low body mass, regressed gonads, white fur, and daily torpor, while others develop only some adjustments or maintain a summer phenotype. Despite comprehensive research, the mechanisms underlying polymorphism of winter phenotype are still unknown. We compared key elements of the hypothalamic thyroid hormone system, as well as the tanycyte architecture in hamsters of both sexes. Individuals presented different responses to short photoperiod characterized either as phenotypes (non-responder, partial-responder and full-responder) or photoresponsive index. We measured the expression of genes coding iodothyronine deiodinase 2 and 3, monocarboxylate transporter 8, thyrotropin-releasing hormone, and somatostatin in 40 individuals and counted the number of immunolabeled tanycyte processes in standardized regions of interest around the third ventricle in 30 individuals. Animals acclimated to short photoperiod presented a downregulation of diodinase 2 and somatostatin and an upregulation of deiodinase 3, as well as a decreased number of tanycyte processes, compared to long photoperiod-exposed individuals. Although phenotypes did not differ in gene expression, the higher the photoresponsive index, the lower was the deiodinase 2 expression and the higher the deiodinase 3 expression. Partial-responders and full-responders had less tanycyte processes than non-responders, and the number of tanycyte processes correlated with the photoresponsive index. Sexes differed neither in their seasonal response, nor hypothalamic gene expression, but females had more tanycyte processes. Our results are in accordance with studies emphasizing the pivotal role of thyroid hormones in seasonal response. We suggest that the whole spectrum of winter phenotypes exists within the population of Djungarian hamsters and that it is reflected also at the level of neuroendocrine regulation. However, the neuroendocrine underpinnings of winter phenotype polymorphism require further investigation.

## Introduction

The Djungarian or Siberian hamster (*Phodopus sungorus*) is a model species for studies of seasonality and photoperiodism [1, 2]. It presents several seasonal changes driven by the length of photoperiod. In summer-like long photoperiod, hamsters are fat, reproductively active, with thin and grey fur, whereas in winter-like short photoperiod (i.e. <13.5h [3]) they develop an energy-efficient winter phenotype with low body mass, regressed gonads, highly insulating white fur, and daily torpor expression [4–7]. However, within a population of Djungarian hamsters, we observe a significant variability in response to short photoperiod. Next to individuals which present a whole suite of winter traits (full-responders), we observe hamsters that develop only some of them, e.g., only white fur or only reduced body mass (partial-responders), and even individuals that maintain summer phenotype throughout the year (non-responders) [7–10]. This phenomenon is called polymorphism of winter phenotype [11, 12] and has been observed in many mammalian species [12–14]. The development of winter phenotype is heritable, and artificial selection can produce responsive or non-responsive breeding lines [15, 16]. However, winter response also depends on photoperiodic history [9, 10, 17–19], maternal programming [20–22], environmental conditions [12, 23–25], and age [26–28]. Although the mechanism of the seasonal response has been comprehensively studied over the last 50 years [1, 29–37], the mechanisms underlying the polymorphism of winter phenotype are still not fully understood [6, 7, 10, 38].

Seasonal changes of the phenotype are driven by photoperiod [4, 30], whereby the development of the winter phenotype requires fully functional seasonal clock mechanisms [39, 40]. Among many factors driving seasonal changes of the phenotype, hypothalamic thyroid hormones levels seem to be crucial [31, 41–44]. The seasonal control of thyroid hormone metabolism within the hypothalamus impacts seasonal changes in reproduction, body mass, and expression of torpor, but not seasonal moulting which is regulated by the prolactin level [45]. The local, hypothalamic control of thyroid hormone levels is achieved by deiodinases [46]. Iodothyronine deiodinase 2 (Dio2) transforms the precursor thyroxine (T4) into the active 3,3′,5-triiodothyronine (T3), whereas iodothyronine deiodinase 3 (Dio3) degrades T3. The expression of *dio3* has been shown to increase in the hypothalamus of short photoperiod-acclimated Djungarian hamsters, whereas *dio2* expression decreased [1, 31, 41, 47]. However, previous studies included only individuals which showed a rather full response to short photoperiod, while individuals presenting other phenotypes were excluded from the experiments [1, 48–50].

Here, we measured expression of genes responsible for the metabolism of hypothalamic thyroid hormone system in the whole spectrum of winter phenotypes, as the polymorphism is assumed to belong to the natural strategy of Djungarian hamster populations [7, 10, 25, 51]. The hypothalamic metabolism of thyroid hormones is predominantly regulated in tanycytes, a specialized glial cell type which forms the wall of the third ventricle and serves as essential interface between the periphery and neuronal tissue [52, 53]. Since their function is considered to depend on the photoperiod and corresponding melatonin signal [54, 55], we also compared the architecture of tanycytes in different winter phenotypes of Djungarian hamsters. To the best of our knowledge this is the first attempt to compare neuroendocrine mechanisms of seasonal response in individuals presenting various responses to short photoperiod. The results of this project will allow a better understanding of the polymorphism of winter phenotype, and ultimately the overall mechanisms of photoresponsiveness.

## Methods

### Animals

All experimental procedures were approved by the Local Committee for Ethics in Animal Research in Bydgoszcz, Poland (decision no 52/2020). Animals were maintained and sacrificed at the Nicolaus Copernicus University in Toruń, Poland, while the laboratory procedures were performed at Ulm University, Germany.

The proportion of non-responders in our outbred colony of Djungarian hamsters *(Phodopus sungorus)* varies between 35 and 75% from year to year, and it is not possible to predict which phenotype will be presented by a certain individual [56]. Therefore, we initially monitored 293 Djungarian hamsters (145 males and 148 females). Animals were maintained in standard laboratory cages (1245; Tecniplast, Italy) with deciduous wood chips as bedding material and paper tubes as nesting material. Since birth, the animals were maintained under a long photoperiod (LP, 16h of light and 8h of darkness, 16:8), at 20 ± 2°C, and fed with standard rodent diet (Labofeed B and H standard, Morawski, Kcynia, Poland). At the age of 10-14 weeks, 269 animals were transferred to a short photoperiod (SP, 8h of light and 16h of darkness, 8:16) to induce winter acclimation response and to determine their winter phenotype. 24 individuals remained in a long photoperiod.

### Selection of experimental animals

To determine the phenotype of individuals, we assessed characteristics related to morphology of animals: body mass changes (*m*_b_), changes in fur colour, as well as torpor use. We did not assess the reproductive status. To measure seasonal changes in body mass, the hamsters were weighed (to the nearest 0.1g; Scout Pro 200, OHAUS) every week or every other week. Fur index was evaluated according to the Figala scale after acclimation to SP [57]. To check whether the hamsters used torpor, they were injected subcutaneously with temperature-sensing transponders (BioTherm 13, Biomark, Boise, ID, USA) in the interscapular region after eight weeks of short photoperiod acclimation. Temperatures were not recorded continuously, but we daily checked the hamsters for torpor use, i.e., torpor was defined as a subcutaneous temperature <30°C. We read the temperature with a remote reader (HPR plus, Biomark, Boise, ID, USA) arbitrarily at different times between circadian time (CT) 2 and 6 on different days to increase the probability of recording all torpor bouts.

After 14 weeks of acclimation, we selected 54 individuals (26 males and 28 females) from the short photoperiod-acclimated hamster cohort. We selected experimental animals randomly, but we wanted to obtain individuals which differ in their phenotype. Since proportion of winter phenotypes varies from year to year, we decided to use 1/3 of grey individuals and 2/3 of white animals, as well as 1/3 of individuals using torpor and 2/3 of individuals not using torpor. This choice should reflect the regular proportion of phenotypes observed in our colony [10, 51, 56]. Another 18 individuals (8 males and 10 females) from the long photoperiod-acclimated hamster cohort was selected randomly as well. The remaining animals were transferred back to the breeding colony. We used 40 animals (10 LP-acclimated and 30 SP-acclimated individuals; 17 males and 23 females) to analyse gene expression by real-time quantitative polymerase chain reaction (qPCR), and 32 individuals (8 LP-acclimated and 24 SP-acclimated individuals; 17 males and 15 females) to measure the tanycyte processes density around the third ventricle by immunohistochemical marking of the intermediate filament vimentin [55]. All animals were killed by cervical dislocation between CT 3.5 and 4.5. Brains were immediately dissected, covered with tissue freezing medium (Tissue-Tek® O.C.T.™, Compound; Sakura Finetek™), frozen on dry ice, and stored at -80°C for later procedures.

In a first analytic approach, we categorized individuals into full-responding, partial-responding, and non-responding phenotypes based on their winter traits. Non-responders (NR) presented fur index ≤ 2, a body mass loss ≤ 5% and no torpor expression, and full-responders (FR) presented fur index ≥ 4, a body mass loss ≥ 10%, as well as torpor expression. All remaining individuals showing only some winter traits (e.g., body mass loss ≥ 10% but grey fur, or white individuals with very low body mass loss) were defined as partial-responders (PR). However, the winter response is not a simple binary trait and the intensity of winter acclimation varies substantially between individuals classified as the same phenotype [10, 15, 58]. Especially partial-responders do not constitute a group of similar individuals but present many different sets of winter traits [51]. Therefore, we used a second analytic approach by quantifying the hamsters’ photoresponsiveness using the metric photoresponsive index (PI) proposed by Lynch et al. [15]. The original quantitative index combines body mass loss (g) and winter moult. These traits were chosen because 1) they are easy and repeatable to measure in many hamsters, 2) the distribution of this index in a population is normal [15], and 3) this index reflects also other aspects of the winter response, i.e., hamsters with high PI undergo gonadal regression and most of them use daily torpor [59]. In this study, we complemented the original equation with torpor expression:

$$PI=\Delta m_{b}+2∙(FI-1)+TI$$

where Δ*m*_b_ was total body mass loss (g), FI was fur index and TI was torpor index. The torpor index was set to 5 to contribute about equally to the PI with other winter traits, since within short photoperiod-acclimated animals median Δ*m*_b_ = 4.1 g, and the median FI = 4. Individuals which used torpor got TI = 5, and individuals which did not use torpor received TI = 0. Since winter traits can develop independently in Djungarian hamsters [10, 15, 60], use of these three traits allowed us to cover whole spectrum of winter response. As long photoperiod-acclimated animals and non-responders might increase their body mass, their photoresponsive index can also be negative.

### Procedures

#### Real-time quantitative polymerase chain reaction (qPCR)

qPCR was performed to compare relative gene expression values between experimental groups. We measured the expression of key genes in the hypothalamic thyroid hormone system: iodothyronine deiodinase 2 (*dio2*), iodothyronine deiodinase 3 (*dio3*), monocarboxylate transporter 8 (*slc16a2*, hereafter called *mct8*), thyrotropin-releasing hormone (*trh*). Moreover, we measured the expression of somatostatin (*srif*) since it is an appropriate non-thyroid indicator of seasonal changes in the hypothalamus [61]. The hypoxanthine phosphoribosyltransferase (*hprt*) served as housekeeping gene. It was shown that *hprt* showed the highest stability, efficiency, and abundance across the tissues of Djungarian hamsters, without photoperiodic changes [62]. Frozen brains were cut to obtain hypothalamic blocks, according to the mouse brain atlas [63]. The hypothalamic blocks were cut from the optic chiasm (Bregma -0.30 mm) to the mammillary bodies (Bregma -2.80 mm). Laterally, the blocks were cut at the hypothalamic sulci and dorsally 3-4 mm from the ventral surface.

To isolate total RNA from the hypothalamic blocks, samples were homogenized in 1000 μl TRI Reagent® (Sigma Aldrich, Germany), and 200 μl of chloroform using a tube with zirconium beads (Biozym, Austria) and a microtube homogenizer (D1030-E, BeadBug; Benchmark scientific, USA). Total RNA was purified with the Crystal RNA MiniKit (Biolab Products, Germany) according to manufacturer instructions, including DNAse treatment (Quiagen, Germany). Purity and quantity of RNA were assessed by determining the optic density photometrically using 260/280 nm and 260/230 nm ratios (NanoDrop ND-2000, Thermo-Fisher Scientific, USA). Mean RNA yield was 763 ng/μL (range: 454-1093 ng/μL, S1 Table, S1 Fig). The RNA integrity was assessed by the Genomics Core Facility of the Faculty of Medicine at Ulm University via automated gel electrophoresis (Agilent 4200 TapeStation System, Agilent Technologies Deutschland GmbH, Waldbronn, Germany) and mean RNA integrity number equivalent (RIN^e^) for our samples was 8.6 (range 8.3-9.0) (S1 Table). Based on the RNA concentration, we calculated the volume needed to get 1 μg of total RNA from each sample and to synthetize cDNA from it using RevertAid H Minus First Strand cDNA Synthesis Kit (Thermo Fisher Scientific, USA) according to manufacturer instruction.

DNA oligonucleotide PCR primers were designed as previously described [44, 64]. All primers (20-25 bp) were designed using the online tool OligoAnalyzer 3.1. The amplicons were cloned into the pGEM1-T or pGEM1-T Easy Vector System (Promega, Madison, USA) according to manufacturer instruction. The cDNA fragments were Sanger-sequenced by the commercial sequencing platform GATC Biotech (Konstanz, Germany). Only the primers that gave single-band amplicons in the presence of RT and that matched the base length of the predicted target were selected (Table 1). In addition, the selected primers yielded 96.1%-115.9% efficiency on RT-qPCR. qPCR measurements were carried out on a Quant StudioTM 3 System (Thermo Fisher Scientific, USA) using Platinum® SYBR® Green qPCR SuperMix-UDG (Invitrogen by Thermo Fisher Scientific, USA). qPCRs were performed separately for each gene of interest on 96-well plates (Fast Gene®96-weel PCR Abi-style, Nippon Genetics, Japan), with two plates per gene. To control for inter-plate variation, we used one sample of a long photoperiod-acclimated hamster as an inter-plate control. To rule out extraneous nucleic acid contamination and primer dimer formation, no-template controls were included in each experiment. To calculate PCR efficiency, we generated a standard curve using eight 1:10 dilutions of the target gene in specific standard plasmids. All samples, no-template control, and inter-plate control were run in technical triplicates and standard plasmids dilutions in duplicates. We used 1 μl of cDNA as template for each reaction and 1 μl of water for no-template control. qPCR was performed with a standard cycling protocol of 2 min at 50°C, then 10 min at 95°C, and 40 amplification cycles with 15 s at 95°C, 15 s at 60°C, and 30 s at 72°C. Subsequently, specificity of each amplification reaction was validated by dissociation curve analysis. The expression of the respective gene of interest was measured relative to the reference gene, *hprt*, and differential gene expression was determined compared to long photoperiod-acclimated animals using the Pfaffl method [65]. The threshold cycle ranges within experimental animals were 19.05-19.92 for *hprt*, 26.48-28.62 for *dio2*, 27.23-36.70 for *dio3*, 23.17-26.34 for *mct8*, 20.28-21.81 for *srif*, and 22.66-26.84 for *trh*. Data were evaluated with the Quant Studio Design and Analysis Software v.1.4.3. (Thermo Fisher Scientific, USA), and then exported to Microsoft Excel files. The amplification efficiency of the qPCR reaction for genes of interests ranged between 96 and 108%. If standard deviation of the technical triplicates exceeded 5%, we excluded the value of the sample. Standard deviation of inter-plate control samples did not exceed 3%.

**Table 1:** Primers used in qPCR analysis.

Target gene	Gene Symbol	Primer sequence Forward 5’ – 3’ Reverse 5’ – 3’	E% (min-max)	Tm °C (Fwd-Rv)	Product length (bp)	Anneal. temp (°C)
*hypoxanthine phosphoribosyl-transferase*	*hprt*	AGTCCCAGCGTCGTGATTAGTGA CGAGCAAGTCTTTCAGTCCTGTC	108.7 115.9	70.9 71.4	140	58
*iodothyronine deiodinase 2*	*dio2*	TGAAGAAACACAGGAGCCAAGAGGA CATTATTGTCCATGCGGTCAGCCA	96.1 98.3	70.8 73.3	111	58
*iodothyronine deiodinase 3*	*dio3*	AGACTTCTTGTGCATCCGCAAGC CACCTCCTCGCCTTCACTGTTG	100.6 106.2	70.6 70.2	94	60
*monocarboxylate transporter 8*	*slc16a2 (mct8)*	GTCCTCTCATTCCTGCTCCTGG GTCCCACCAGCTCAAATGCAATG	105.7 105.8	68.4 71.5	151	60
*thyrotropin-releasing hormone*	*trh*	CTCACAGACGCCCGATGCTG GAGTCACTGCATCCTCCTCCTG	97.3 108.7	61.0 59.0	124	60
*somatostatin*	*srif*	GAAGTCTCTGGCGGCTGCTG CAGCCTCATTTCATCCTGCTCCG	96.7 100.7	69.8 72.7	103	60

melting temperature (Tm), base pair (bp)

#### Immunohistochemistry (IHC)

The visualization of tanycyte processes around the third ventricle of the hypothalamus was achieved by an immunohistochemical (IHC) fluorescence staining against the intermediate filament vimentin. Brains were moved from -80° to -20°C one day before coronal sectioning between Bregma 0.2 and -2.8 mm [63]. 20 μm slices were mounted on poly-L-lysine slides (Polysine™ adhesion microscope slides, Gerhard Menzel GmbH, Braunschweig, Germany) and stored at -20°C until further use. The slices were fixed in 4% paraformaldehyde (PFA) for 60 min and then washed several times in phosphate buffered saline (PBS) with 0.3% triton. Next, the slices were covered with 5% normal horse serum in PBS + triton (Vector Laboratories, USA) as blocking solution for 1 hour. Afterwards, the slices were incubated with the polyclonal primary rabbit anti-vimentin antibody in a dilution of 1:500 (172 002, Synaptic System, Germany) at room temperature overnight. Immunoreactivity against vimentin was visualized using a secondary donkey anti-rabbit IgG antibody conjugated with Alexa Flour 488 in a 1:300 dilution (A-21206, Thermo Fisher Scientific, USA). A nuclear counterstain was achieved by adding 4,6-diamidino-2-phenylindol (DAPI) in a 1:1000 dilution (D9542, Sigma-Aldrich, Saint Louis, USA). The slices were incubated in darkness for 3 hours. To determine specificity of staining and identify false-positive staining reactions, we used negative staining control procedure in which we did not cover slides with primary antibodies. All slices were covered with MOWIOL (Merc, Germany) and a coverslip and stored in darkness at 4°C.

**Fig 1:**
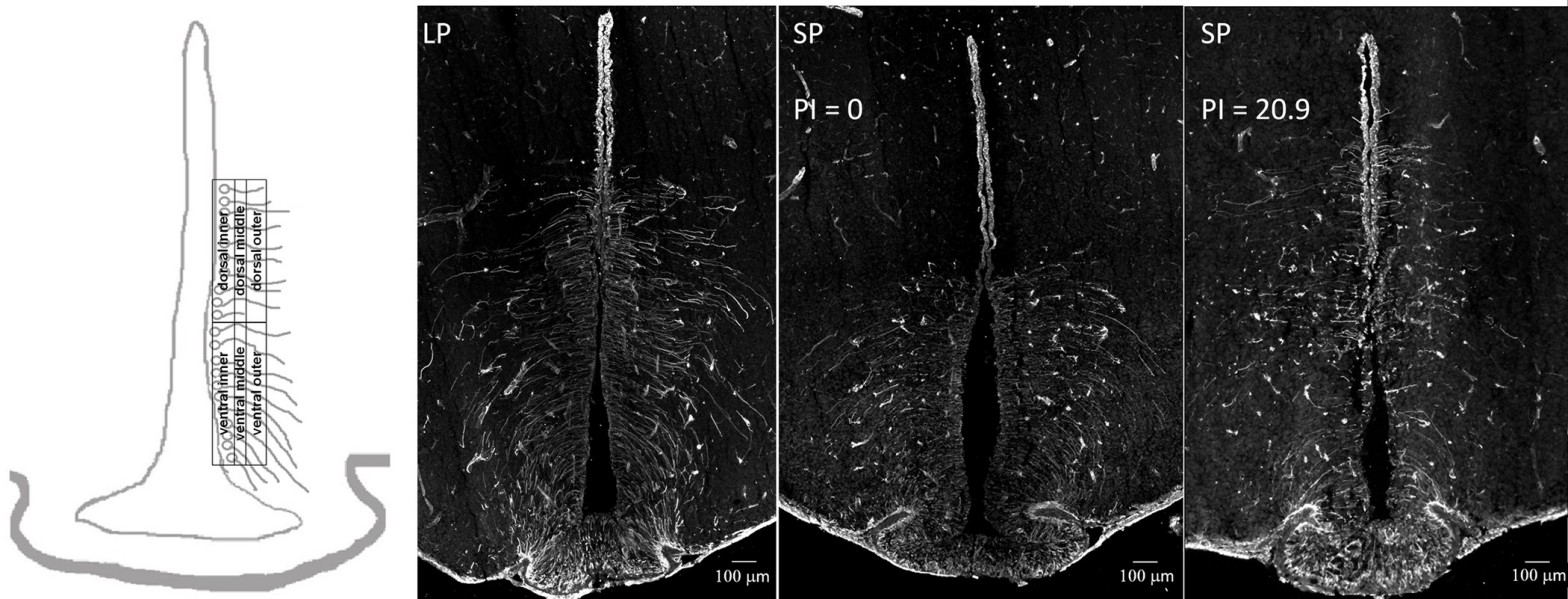
Schematic representation of regions of interest (ROI) for the image analysis with the third ventricle as orientation structure. Size of each rectangle: half of the height of the tanycyte part of the third ventricle and 100 μm width. Immunohistochemical visualization of tanycytes via the fluorescent marking of the intermediate vimentin in the coronal sections of the mediobasal hypothalamus of representative Djungarian hamsters exposed to long photoperiod (LP) and short photoperiod (SP) presenting a different photoresponsive index (PI). The number of tanycyte processes stained against vimentin is higher in individuals with a low PI than in individuals with high PI.

The slices were photographed using a microscope camera (Leica DFC 3000 G; Leica Microsystems, Germany) attached to an automated, upright fluorescence microscope system (Leica DM 6000 FS, Leica Microsystems, Germany) under 100 × magnification. Images were taken using the same settings for all samples (Leica Application Suite X. Ink 3.72.22383; Leica Microsystems, Germany), and analysed using Image J software v. 2.0. (NIH, Bethesda, MD, USA). To compare the seasonal plasticity of the tanycyte architecture between individuals, we standardized the area around the third ventricle as orientation structure to obtain comparable regions of interests (ROI). Therefore, we divided the third ventricle into an ependymal and a tanycyte part. In the former, the ventricular wall does not consist of tanycytes but ependymal cells, while in the latter, the ventricular wall is formed by tanycyte somata, sending their processes to the hypothalamic tissue. The tanycyte part was further divided into dorsal and ventral part, and then into rectangles with a size of half the height of the tanycyte part of the third ventricle and a width of 100 μm. The long side of the inner rectangles (dorsal and ventral) was aligned to the ventricular wall, the long side of middle rectangles was aligned to the long side of the inner rectangles, and the long side of the outer rectangles was aligned with the long side of the middle rectangles (Fig 1). We analysed twelve rectangles in total (six per left and six per right side). In the statistical analysis, data from left and right sides were averaged, so we compared data from six ROIs (three dorsal and three ventral ones). Using the grid formed by the respective ROI rectangles (Fig 1), we manually counted immunostained processes intersecting the grid lines. This method has been used before [54] and gives the reliable information about tanycyte abundance in the medial hypothalamus. We investigated one slice per individual, which was chosen based on the shape of the third ventricle characteristic for the level of the mediobasal hypothalamus, as well as the comparability between individuals. Tanycytes in the median eminence were so densely packed that counting was impossible; therefore, our data include mostly alpha tanycytes. However, we counted all processes above median eminence and believed it also included beta-tanycytes projecting to the arcuate nucleus. All measurements were done by the same person (APP), who was blind to treatment.

#### Statistical analysis

Eventually, we obtained data from 70 individuals (40 for qPCR and 30 for IHC). All statistical analyses were performed in R (RStudio v. 1.0.153 [66]). Data did not meet assumptions for parametric tests, so we used their nonparametric equivalents. First, we used a categorical approach to test the effect of phenotype (full-responding, non-responding, partial-responding) on gene expression and number of tanycyte processes. We used the Kruskal-Wallis test (package FSA version 0.9.5 [67], function: kruskal.test) and post hoc Dunn’s test with p-values adjusted with the Benjamini-Hochberg method for multiple comparisons (package FSA [67], function: dunnTest.). Due to a low number of full-responding and non-responding individuals, as well as an unequal distribution of sexes, this analysis did not include the effect of sex. Second, we tested the effect of the metric PI on gene expression and tanycyte processes number. We used the Scheirer–Ray–Hare test with two-way factorial design (package rcompanion v. 2.4.21 [68], function: scheirerRayHare) to compare photoperiod regimes and sexes. All models included the effect of photoperiod, sex, and the interaction of both factors.

The effect of PI on gene expression and number of tanycyte processes was tested using Spearman’s rank test (package stats v. 4.2.3 [66], function: cor.test, method= "spearman"). If there was an effect of sex on a measured trait, we correlated data from each sex separately. Differences or correlations with P-values <0.05 were considered as significant, while correlation coefficients of <0.3 were considered as weak, between 0.3 and 0.5 as medium, and >0.5 as strong correlation. Data are presented on scatter plots and boxplots. Boxes cover the 25^th^ to 75^th^ percentiles, while horizontal lines within boxes indicate medians. Whiskers (error bars) above and below the box indicate the 90^th^ and 10^th^ percentiles.

## Results

### Photoresponsiveness of the animals

Animals from different photoperiod regimes did not differ before the start of the experiment (median *m*_b_ 32.2 g, t_(1, 70)_ = -0.43, P = 0.661). The long photoperiod-acclimated individuals weighed 32.4 g, and during acclimation period their body mass change ranged from -1,9 g to 6.2 g (median = 0.8 g). Their fur index was 1 and they had not used torpor. Within this group, males more often increased body mass, whereas females maintained a stable body mass throughout the experiment, but this difference did not reach significance (t_(1, 15)_ = 1.75, P = 0.10).

**Fig 2:**
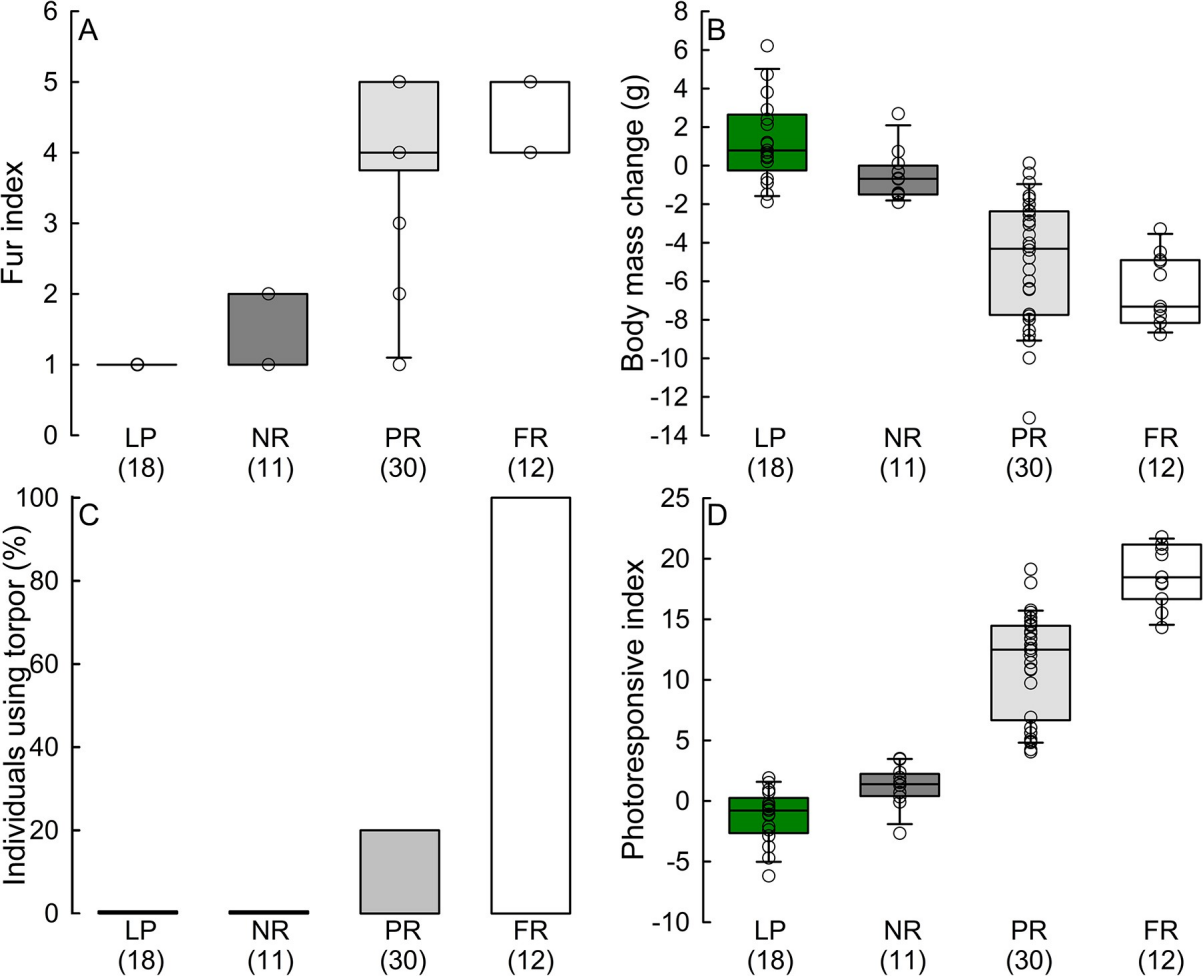
Effect of winter phenotype on fur index (Panel A), body mass change (Panel B), individuals using torpor (Panel C), and photoresponsive index (Panel D) presented by Djungarian hamsters. LP – long-photoperiod acclimated individuals, NR – non-responding individuals, PR – partial- responding individuals, FR – full-responding individuals. Differences between phenotypes are always significant because of the grouping criteria. Numbers in brackets indicate sample size.

According to photoresponsive categories, we obtained 11 full-responders (5 in qPCR and 6 in IHC analysis), 12 non responders (7 in qPCR and 5 in IHC analysis), and 30 partial-responders (18 in qPCR and 12 in IHC analysis). Although we did not do formal statistical analysis, we observed similar number of males and females presenting the full-responding (6 and 5, respectively), and the non-responding phenotype (6 and 6). Phenotypes differed in winter adjustments. Fur index was high in full-responders (fur index = 5) and partial-responders (fur index = 4), while fur index of non-responders was 1 (Fig 2A). Full-responders lost 7.3 g, partial-responders 4.3 g, and non-responders 0.7 g (Fig 2B). Moreover, all full-responders used torpor, but 20% of partial-responders used torpor (Fig 2C). This resulted in different PI (*H*_(3, 70)_ = 58.29, P <0.001). Photoresponsive index was 18.5 in full-responders, 12.5 in partial-responders, while non responders had PI = 1.4 (Fig 2D).

**Fig 3:**
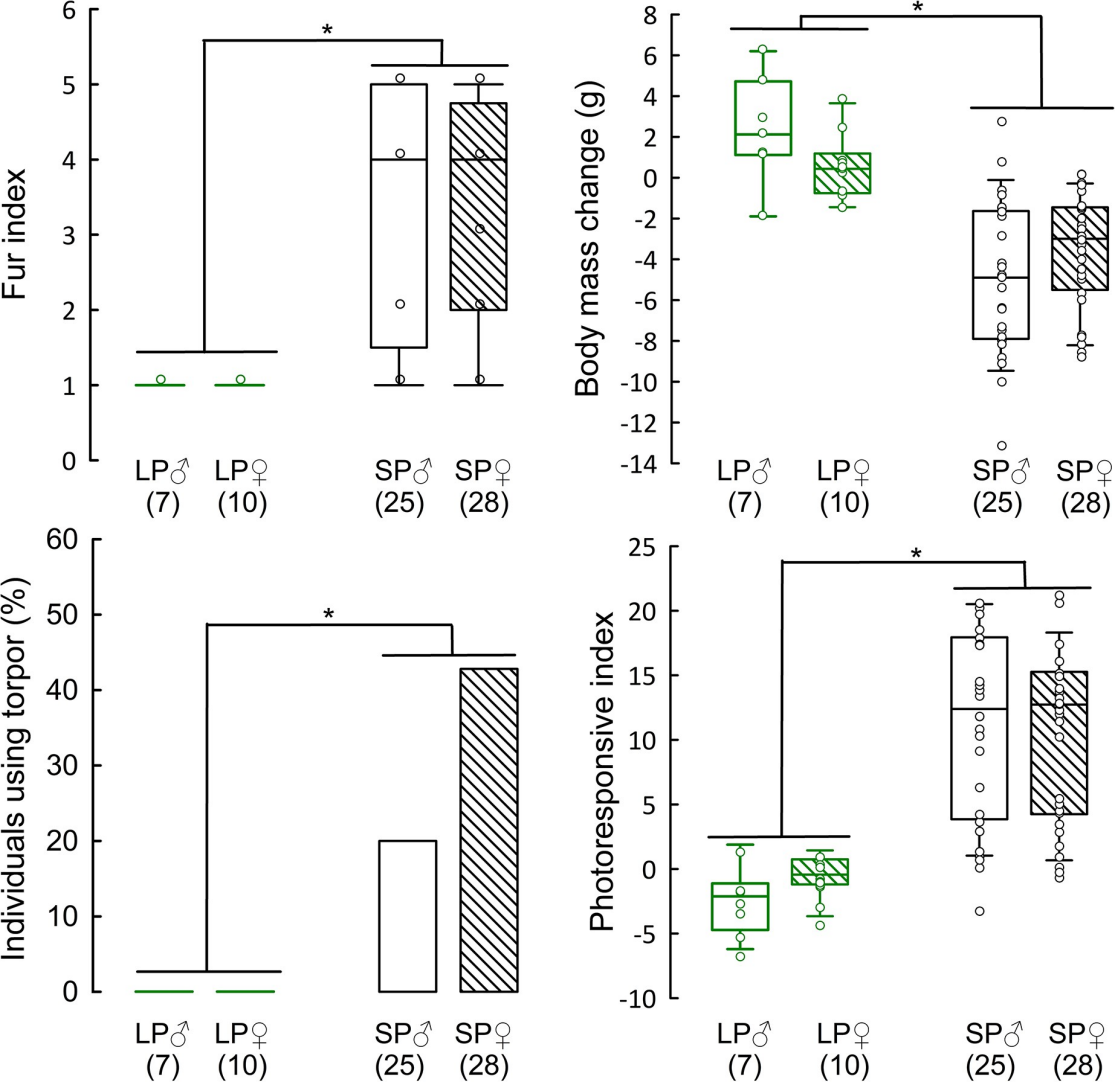
Effect of photoperiod and sex on fur index (Panel A), body mass change (Panel B), individuals using torpor (Panel C), and photoresponsive index (Panel D) of Djungarian hamsters. LP – individuals acclimated to long photoperiod, SP – individuals acclimated to long photoperiod. Numbers in brackets indicate sample size. Scheirer–Ray–Hare showed effect of photoperiod but no effect of sex. *P <0.05.

When we analysed the whole population without separation into phenotypes, we observed the whole spectrum of photoresponsiveness. Fur index ranged from 1 to 5 (median = 4), while body mass loss was 4.2 g (range -13.1 g to 2.7 g). Sex did not affect these seasonal changes (fur index: *t*_(1, 53)_ = -0.37, P = 0.71; body mass change *t*_(1, 53)_ = 1.55, P = 0.12; Fig 3A, 3B). 32% of individuals (17 out of 53) used torpor and we found a trend towards a higher torpor use in females (43%) than in the males (20%) (Fisher exact test, P = 0.088); Fig 3C). Finally, median PI was 12.4, but it ranged from -2.7 to 21.8 (Fig 3D). Sex did not affect PI (t_(1, 53)_ = -0.30, P = 0.76) and the median PI in males was 12.4, ranging from -2.7 to 21.2, while the median PI in females was 12.7 with a range from -0.1 to 21.8. (Fig 3D).

#### Genes expression

Expression of *trh*, and *mct8* did not differ between phenotypes (*trh*: H_(3, 39)_ = 4.10, *P* = 0.25, *mct8*: H_(3, 39)_ = 1.44, *P* = 0.69), while expression of *dio3* and *srif* did (*dio3*: H_(3, 39)_ = 10.14, *P* = 0.01, *srif*: H_(3, 39)_ = 10.195, *P* = 0.02). Long photoperiod-acclimated animals had a lower *dio3* expression than partial-responders (z_(3, 39)_ = -3.13, P = 0.002), while *srif* expression was higher in long than in short photoperiod (long photoperiod vs. full-responders: z_(3, 39)_ = -2.08, P = 0.641; vs. partial-responders: z_(3, 39)_ = -1.98, P = 0.05; vs. non-responders: z_(3, 39)_ = -3.02, P = 0.002) (S2 Fig). We also found that the expression of *dio2* tended to differ between phenotypes, but this difference did not reach a significance level (*dio2*: H_(3, 39)_ = 6.79, *P* = 0.08).

**Fig 4:**
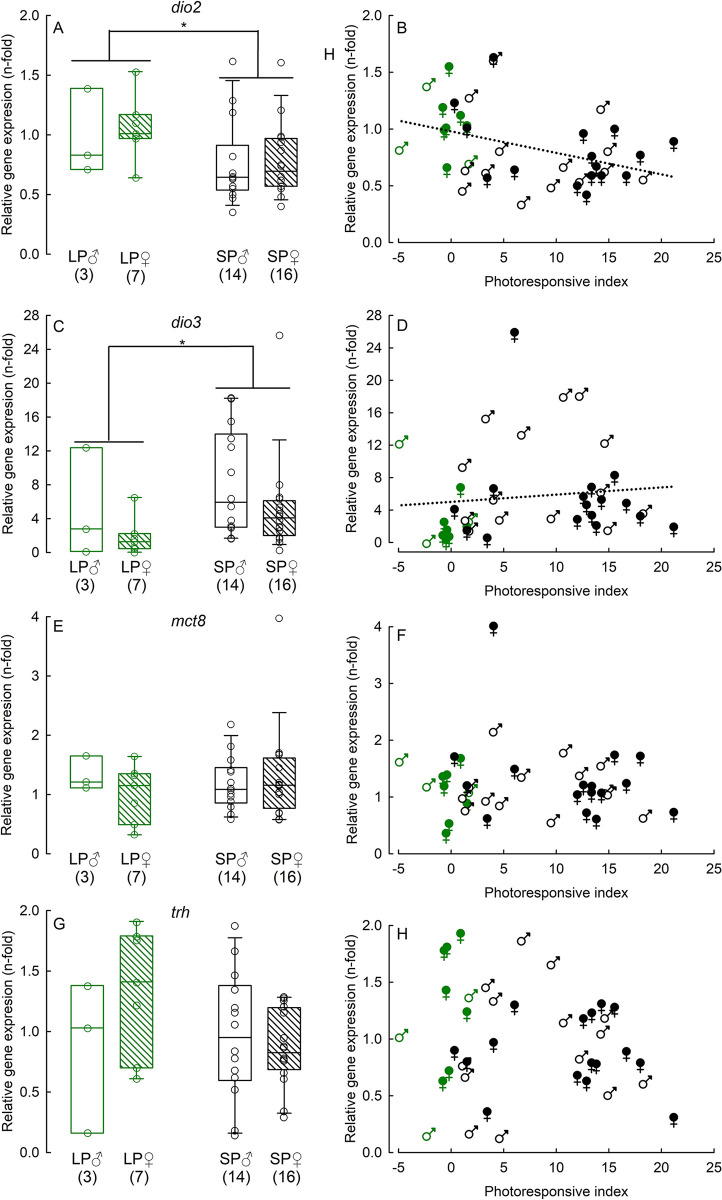
Relative gene expression of iodothyronine deiodinase 2 (*dio2*; Panels A, B), iodothyronine deiodinase 3 (*dio3*; Panels C, D), monocarboxylate transporter 8 (*mct8*; Panels E, F), and thyrotropin-releasing hormone (*trh*; Panels G, H) in long photoperiod-acclimated (LP), and short photoperiod-acclimated (SP) Djungarian hamsters. Panels A, C, E, G: Effect of photoperiod and sex on gene expression. Numbers in brackets indicate sample size. *P <0.05. Panels B, D, F, H: Spearman rank correlation between photoresponsive index (PI) and gene expression. Significant correlation is presented as regression line in whole population since there was no effect of sex on genes expression.

Independent of phenotype, expression of *dio2* and *dio3* did not differ between sexes (*dio2*: H_(1, 39)_ = 0.52 *P* = 0.47; *dio3*: H_(1, 39)_ = 2.61 *P* = 0.10), but it was related to photoperiod (*dio2*: H_(1, 39)_ = 5.86, *P* = 0.015; *dio3*: H_(1, 39)_ = 5.56 *P* = 0.018) (Fig 4A and 4C). *Dio2* was downregulated and *dio3* was upregulated in short photoperiod-acclimated animals compared to long photoperiod-acclimated ones. Moreover, we found a medium negative correlation between photoresponsive index and *dio2* expression (r_s_ = -0.37, *P* = 0.018) and a medium positive correlation between photoresponsive index and *dio3* expression (r_s_ = 0.31, *P* = 0.047) (Fig 4B and 4D). Expression of *mct8* and *trh* were neither affected by sex (H_(1, 39)_ = 0.16, P = 0.691 and H_(1, 39)_ = 0.01, P = 0.753, respectively), nor by photoperiod (H_(1, 39)_ = 0.01, P = 0.933 and H_(1, 39)_ = 2.20, P = 0.137, respectively) (Fig 4E and 4G). It did not correlate with the photoresponsive index either (*mct8*: r_s_ = -0.05, *P* = 0.74; *trh*: r_s_ = -0.15, *P* = 0.34) (Fig 4F and 4H).

**Fig 5:**
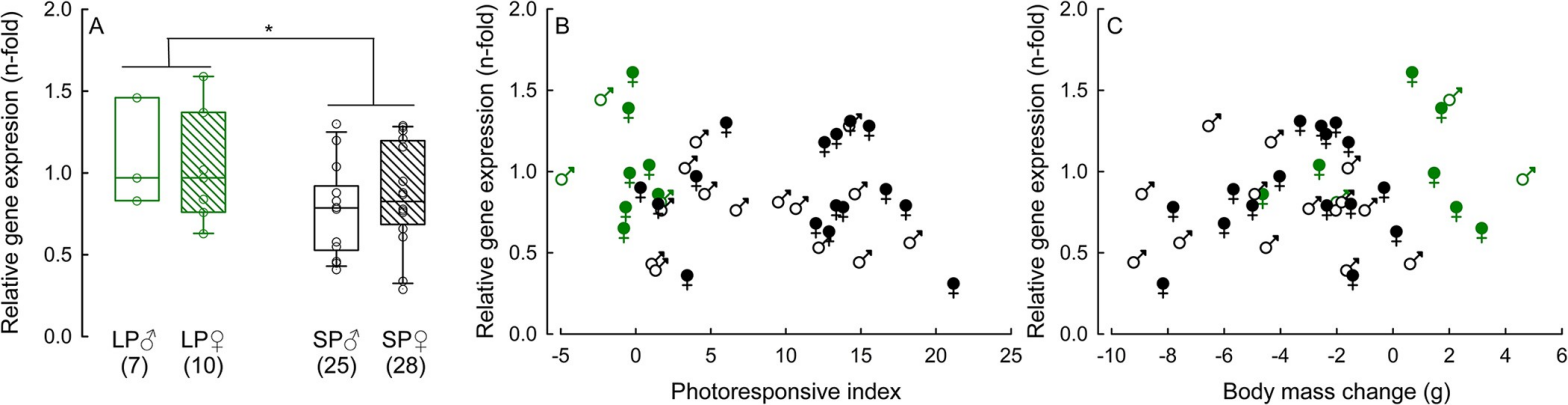
Relative gene expression of somatostatin (*srif*) in long photoperiod-acclimated (LP) and short photoperiod-acclimated (SP) Djungarian hamsters. Panel A: Effect of photoperiod and sex on *srif* expression. Numbers in brackets indicate sample size. There was only an effect of photoperiod. *P <0.05. Panel B: Spearman rank correlation between photoresponsive index and *srif* expression. Panel C: Spearman rank correlation between body mass change after 16 weeks of acclimation and *srif* expression. Both correlations were not significant.

Sex did not affect the expression of *srif* (H_(1, 39)_ = 0.41, *P* = 0.52), but *srif* was downregulated in short photoperiod-acclimated animals relative to long photoperiod-acclimated animals (H_(1, 39)_ = 8.27, *P* = 0.004; Fig 5A). We neither found a correlation between photoresponsive index and *srif* expression (r_s_ = -0.27, *P* = 0.09; Fig 5B) nor between body mass change and *srif* expression (r_s_ = 0.12, *P* = 0.44; Fig 5C).

#### Immunoreactivity

The number of tanycyte processes was similar in the dorsal and ventral ROIs, but it decreased with increasing distance from the ventricular wall, and the number of processes in the outer ROIs was half of that in the inner ROIs. Phenotypes differed in the number of tanycytes processes in all dorsal ROIs (dorsal inner ROI: H_(3, 29)_ = 14.43, *P* = 0.002; dorsal middle ROI: H_(3, 29)_ = 15.96, *P* = 0.001, dorsal outer ROI: H_(3, 29)_ = 9.70, *P* = 0.021) and in two ventral ROIs (ventral inner ROI: H_(3, 29)_ = 13.03, *P* = 0.004; ventral middle ROI: H_(3, 29)_ = 8.32, *P* = 0.040). In these ROIs, long photoperiod-acclimated hamsters and non-responders had significantly more tanycytes processes than full-responders and partial-responders (Table 2). Phenotype did not affect number of tanycytes processes in ventral outer ROI (H_(3, 29)_ = 4.28, *P* = 0.23). Moreover, number of tanycytes processes did not correlate with photoresponsive index within phenotypes, except two ROIs in non-responders (Table 3).

**Table 2:** Effect of phenotype on the number of tanycytes processes counted in different regions of interest around third ventricle in Djungarian hamsters.

		Dorsal Inner	Dorsal Middle	Dorsal Outer	Ventral Inner	Ventral Middle	Ventral Outer
	N						
LP	7	34		23	35	28	17
NR	5	34	30	22	32	26	15
PR	12	25	21	15	20	16	12
FR	6	24	19	15	22	16	12
		*	*		*	*	

LP – long photoperiod acclimated individuals, NR – non responding individuals, PR – partial responding individuals, FR – full responding individuals.

*Significant difference between phenotypes. Data presented as median.

**Table 3:** Result of Spearman’s rank correlation test. Correlation between number of tanycyte processes in different regions of interest around third ventricle and photoresponsive index in different winter phenotypes of Djungarian hamsters.

	Photoresponsive index vs. no. tanycyte processes							

	LP		NR (N = 5)		PR (N = 12)		FR (N = 6)	
	r_s_	*P*	r_s_	*P*	r_s_	*P*	r_s_	*P*
Dorsal inner	-0.57	0.166	0.69	0.205	-0.24	0.272	0.18	0.724
Dorsal middle	-0.13	0.792	0.78	0.118	-0.06	0.777	-0.12	0.819
Dorsal outer	0	1	0.76	0.129	0.19	0.369	-0.58	0.222
Ventral inner	-0.20	0.666	**0.89**	**0.039**	-0.28	0.210	-0.59	0.213
Ventral middle	0.14	0.711	**0.92**	**0.024**	0.015	0.944	-0.19	0.719
Ventral outer	0.40	0.381	0.69	0.192	0.031	0.889	-0.31	0.539

LP- long photoperiod acclimated individuals, NR-non responding individuals, PR – partial responding individuals, FR- full responding individuals. Statistically significant correlations are bold.

Independent of phenotypes, photoperiod affected the number of tanycyte processes in both inner (dorsal inner ROI: H_(1, 29)_ = 6.40, *P* = 0.011; ventral inner ROI: H_(1, 29)_ = 9.64, *P* = 0.001) and middle ROIs (dorsal middle ROI: H_(1, 29)_ = 8.59, *P* = 0.003; ventral middle ROI: H_(1, 29)_ = 4.85, *P* = 0.027), whereby the number of tanycytes was lower in short photoperiod- than in long photoperiod-acclimated animals. Males and females differed in the number of tanycyte processes in the ventral ROIs only (ventral inner ROI: H_(1, 29)_ = 4.93, *P* = 0.026; ventral middle ROI: H_(1, 29)_ = 4.05, *P* = 0.044, ventral outer ROI: H_(1, 29)_ = 5.41, *P* = 0.020) with females having more tanycyte processes than males (Table 4).

**Table 4:** Effect of photoperiod and sex on the number of tanycytes processes counted in different regions of interest around third ventricle, in Djungarian hamsters.

			Dorsal Inner	Dorsal Middle	Dorsal Outer	Ventral Inner	Ventral Middle	Ventral Outer
		N						
LP	♂	4	35	28	18	32	24	16
	♀	3	33	33	18	39	32	17
SP	♂	22	24	22	16	20	14	10
	♀	12	28	22	17	29	23	15
			*	*		* #	* #	#

* LP – long photoperiod acclimated hamsters, SP – short photoperiod acclimated hamsters.

♂ - males; ♀- females

*significant difference between photoperiod regimes

# significant difference between sexes. Data presented as median.

When we analysed the whole set of experimental animals, the number of tanycyte processes was negatively correlated with the photoresponsive index and positively correlated with the seasonal body mass change in all ROIs except ventral outer ROI (Table 5).

**Table 5:** Result of Spearman’s rank correlation test. Correlation between number of tanycyte processes in different regions of interest around third ventricle and photoresponsive index or body mass change.

	Photoresponsive index vs. no. tanycyte processes		Body mass change (%) vs. no. tanycyte processes	

	r_s_	*P*	r_s_	*P*
Dorsal inner	**-0.63**	**< 0.001**	**0.63**	**< 0.001**
Dorsal middle	**-0.66**	**< 0.001**	**0.61**	**< 0.001**
Dorsal outer	**-0.44**	**0.013**	**0.50**	**0.004**
Ventral inner	**-0.63**	**< 0.001**	**0.60**	**0.001**
Ventral middle	**-0.43**	**0.027**	**0.43**	**0.012**
Ventral outer	-0.28	0.012	0.27	0.146

Significant correlations are bold.

## Discussion

The mechanisms of seasonal responsiveness have been comprehensively studied in Djungarian hamsters (reviewed [2]), but it has never been examined in the context of the variability in winter response until now. Here, we compared key elements of thyroid hormone metabolism and tanycyte architecture in the hypothalamus of male and female Djungarian hamsters presenting different winter phenotypes. Experimental animals showed the whole spectrum of winter response, which was reflected in a huge variability in gene expression and complexity of tanycyte architecture around the third ventricle. Although phenotypes did not differ in gene expression, the photoresponsive index was related to expression of *dio2* and *dio3* as well as to the number of tanycyte processes, which emphasizes the pivotal role of thyroid hormones in the development of winter phenotype. Sexes neither differed in winter response nor in seasonal changes in the hypothalamus, which suggests that polymorphism of winter phenotype is independent on sex.

### Photoresponse and seasonal changes

Seasonal changes in phenotype are orchestrated by a complex neuroendocrine network [45, 53] which is regulated by photoperiod. We found that *dio2* was downregulated and *dio3* was upregulated in short photoperiod-acclimated individuals compared to long photoperiod-acclimated ones (Fig 4A and 4C). These photoperiodic changes in expression of deiodinases would lead to a decrease of thyroid hormone levels the in hypothalamus and ultimately to the development of the individual winter phenotype [41, 43]. In accordance with this, we found that the intensity of the winter response was directly related to the level of deiodinases expression. The stronger the winter response, i.e., the higher the photoresponsive index, the lower was the level of *dio2* and the higher was the level of *dio3* expression (Fig 4B and 4D). Such a correlation between the expression of deiodinase-encoding genes and the winter response suggests that differences in hypothalamic thyroid hormone metabolism determine polymorphism of winter phenotype in Djungarian hamsters.

The highest expression of *dio2* and *dio3* occurs in astrocytes and tanycytes lining the third ventricle and the median eminence [46]. Studies on tanycyte morphology suggest that seasonal changes in the architecture of tanycytes around the third ventricle might be the crucial component of the seasonal response in vertebrates [54, 55]. Here, we observed fewer vimentin-stained tanycyte processes in short photoperiod-acclimated animals compared to long photoperiod-acclimated animals (Table 4). It is consistent with previous research showing a short photoperiod-related decrease in the expression of vimentin, neural cell adhesion molecule, glial fibrillary acidic protein, nestin, and polysialic acid [54, 55, 69, 70], which leads to the rearrangement in the length and number of tanycyte processes. The winter reduction of tanycyte processes is regulated by seasonal changes in the level of melatonin [55], but the purpose of this reduction is still not known. Here, we found that partial- and full-responders had significantly less tanycyte processes than non-responders and long photoperiod-acclimated hamsters (Table 2), and that intensity of winter response (PI) negatively correlated with number of tanycyte processes (Table 5), which means that a stronger winter response was related to the decrease of the complexity of tanycyte architecture around the third ventricle. It still remains questionable whether the reduction of tanycytes is a precisely regulated process which might have evolved to reduce unnecessary neural connections to optimize energy efficiency on central nervous system level or whether it might be the side effect of inhibiting the maintenance of a reproductively active and highly energy-demanding summer phenotype.

We did not find differences in *mct8* and *trh* expression between photoperiod regimes (Fig 4E–4H). Among thyroid hormone transporters, Mct8 is probably the best studied because of its high affinity and transport rate for T3 [71]. In mice, *mct8* is highly expressed in TH-sensitive brain regions, such as in the cerebral cortex, hippocampus, and also in the hypothalamus [72]. In the latter, a strong expression was detected in the median eminence and in tanycytes lining the 3V, while moderate expression levels were observed in the paraventricular and periventricular nuclei. Structure and function of *mct8* are evolutionary highly conserved among vertebrates [73], and also in the hamster hypothalamus expression is by far strongest in tanycytes projecting in the ventromedial nuclei, the arcuate nuclei, and the median eminence [31, 43]. Although expression of *mct8* has been shown to change seasonally and to increase in short photoperiod [31, 43], it also depended on age, week of acclimation, and photoperiodic history [31, 74–76]. Earlier studies revealed that the expression of *mct8* did no longer change after 14 weeks acclimation to short photoperiod [76] or when animals were acclimated to long photoperiod for over 25 weeks [43]. In our study, SP hamsters had experienced approximately 14 weeks of long photoperiod followed by 14 weeks of short photoperiod during their life, while LP hamsters had experienced 28 weeks of long photoperiod only. Thus, although the hamsters were sacrificed with comparable age, the absolute amount of lifetime in a certain photoperiod differed substantially, which might explain the lack of difference in *mct8* expression. Apart from that, expression of *mct8* was also not related to the intensity of seasonal response. One could expect the opposite since non-responders for re-acclimation to short photoperiod did not change *mct8* expression seasonally, while responders did [76]. However, activity of this bidirectional T4/T3 transporter should always be considered in the context of thyroid hormone availability. Expression of *trh* did not change seasonally but was rather related to the metabolic/energy status of an individual [31, 42, 76], and our results support this (Fig 4G). However, the development of winter traits is considered as energy saving strategy [77–79]. Since, winter phenotypes should differ in energy efficiency, we might have expected that animals with more intense winter responses and thus a higher PI would have lower levels of *trh*. However, such relationship has not been found. It suggests that different winter phenotypes of Djungarian hamsters did not differ in energy efficiency in short photoperiod, which is in accordance with our previous studies on metabolism and foraging activity in this polymorphic species [10, 56, 60]. Nevertheless, we should remember that the level of *trh* is regulated by thyroid hormones in hypophysiotropic neurons of the paraventricular nuclei only, while we used whole hypothalamus samples which might have blurred the exact pattern on *trh* expression.

Unexpectedly, we found that somatostatin expression was downregulated in short photoperiod-acclimated hamsters (Fig 5), while previous research showed a short photoperiod-related increase in *srif* levels [43, 48, 75, 76]. The primary role of somatostatin is the inhibition of the growth axis, and its increase in short photoperiod is directly or indirectly responsible for the seasonal decrease of body mass in hamsters [80]. Therefore, one could expect increased *srif* levels in more photoresponsive individuals. However, *srif* expression correlated neither with PI nor with body mass change (Fig 5). Our results might be partially explained by the broad range of seasonal changes in the hamsters’ body mass (Figs 2 and 3), as some individuals did not lose body mass, while others reduced it by over 20%. This natural variability between short photoperiod-acclimated individuals from the same cohort makes it difficult to generalize seasonal changes in gene expression, but emphasizes that distinct, individual gene expression patterns underlie the variable energy saving strategies employed by Djungarian hamsters. However, the *srif* level was reported to change seasonally in the posterior arcuate nuclei only [81]. Thus, the whole hypothalamus sample of the present study might again be responsible for the unclear results, as the local increase of *srif* in short photoperiod was not recognised due to potentially decreased levels in other hypothalamic structures. We are aware that the methodology used here is not the gold standard for neuroendocrine studies, but this experiment focussed on different winter phenotypes of hamsters for the first time. Further investigation is needed to unravel the mechanism of winter phenotype polymorphism in this species.

### Effect of sex

In this study, we used animals of both sexes, but did not find differences in the photoresponsiveness between males and females. They showed similar changes in fur colour and body mass, which resulted in an equal photoresponsive index (Fig 2). On the one hand, 12 out of 17 torpor expressing animals were females, which is consistent with our recent study showing that more full-responders (animals with all winter traits) were observed in this sex [51]. On the other hand, 10 out of 18 animals with the lowest PI (< 5) also were females, showing non responding animals of both sexes. Also, males and females were equally distributed in all phenotypes. Gene expression was not affected by the sex of the animals, but we found that females had an overall higher number of tanycyte processes, both in long and short photoperiod-acclimated animals. Although, it was shown that the vimentin level is independent of seasonal sex steroid changes [54], sex-specific differences in tanycyte architecture cannot be excluded. Sex differences in the neuroendocrine mechanism of seasonality have been usually studied in the context of reproductive activity (reviewed [82]), and expression of kisspeptin or *rfrp* was sexually dimorphic, at least in some hypothalamic nuclei [53]. By contrast, studies on hypothalamic thyroid hormone metabolism focused mostly on males [31, 43, 75]. In a recent review, the sex effect on the activity of hypothalamus-pituitary-thyroid axis was summarized. Authors described no sex difference in *trh* expression in the paraventricular hypothalamic nuclei and in TRH-induced release of thyroid stimulating hormone [83], but this review did not include seasonal or photoperiodic species. Apparently, our results are the first direct comparison of hypothalamic thyroid hormone metabolism between male and female Djungarian hamsters. The effect of steroid hormones on thyroid axis and also seasonal dimorphism in particular nuclei of the hypothalamus deserve further study.

### Limitations of the study

The variability of results observed in this study must be considered. Based on winter traits, we characterized hamsters via categorical phenotypes or by calculating the metric photoresponsive index. Variability in winter response was reflected in the variability of hypothalamic gene expression within phenotypes. We did not find the effect of phenotype on gene expression, which was probably because some non-responding individuals presented genes expressions similar to full-responders. In contrast, others were comparable to long photoperiod-acclimated animals. It was previously shown that some non-responders have malfunctioned circadian clock and are insensitive to changes in photoperiod length [59, 84]. Non-responders that did not differ from long photoperiod-acclimated animals most probably presented this phenotype. However, some non-responders had a regular activity of the circadian clock [59, 84, 85], and we showed that some individuals which do not develop winter traits might present a typical short photoperiod metabolism of thyroid hormones in the hypothalamus (Fig 4). Together with the existence of a whole spectrum of partial-responding individuals with an intermediate winter response, the above-mentioned considerations suggest that polymorphism of winter phenotype might have various origins and that photononresponsiveness might develop on different levels of the neuroendocrine network responsible for photoperiodic changes.

Moreover, the variability of photoresponsiveness within phenotypes suggests that, as we previously showed, the development of winter traits is a continuum, and hamsters of a certain cohort present the whole spectrum of phenotypes [10, 51]. Therefore, we proposed the objective quantitative measure of winter response, i.e., the photoresponsive index. This index combines all winter characteristics and correlates well with neuroendocrine gene expression changes (Fig 4) and tanycytes’ architecture (Table 5). However, this index also has limitations since it mixes traits regulated independently (e.g., body mass change and fur index) and numerical variables (body mass) with categorical ones (torpor). Body mass change is presumably the most reliable factor in the characterization of the winter response since it shows a similar correlation with a number of tanycytes’ processes as PI (Table 5). It is also more objective and easier to asses than fur index and torpor use. Finally, using torpor as a binary variable simplifies the picture of torpor use in hamsters. Although the PI calculated here has some pitfalls, it remains a valuable metric for describing winter response and can be used as a reference in further studies.

In the present study, we did not gather information about reproductive status of the experimental animals, while modulation of reproductive activity throughout the year is one of the main purposes of photoperiodic changes in hypothalamic thyroid hormone levels [41, 42]. Although we cannot draw a final conclusion about the gonadal activity of the hamsters used in this experiment, torpor use, and body mass loss [2] are reliable predictors of gonadal regression. The expression of deiodinases and the number of tanycyte processes were correlated with photoresponsive index and since they mirror the reproductive status in Djungarian hamsters [86], we assume that data collected here reflects all aspects of seasonal acclimation.

## Conclusion

Seasonal changes in phenotype are mainly regulated by photoperiod [4, 87], but the development of winter traits requires a fully functional photoperiodic neuroendocrine network comprising several regulatory levels and different neuroendocrine pathways [40]. We found that winter phenotypes of Djungarian hamsters differed in the activity of the hypothalamic thyroid hormone system as well as architecture of tanycyte processes around the third ventricle. These results support the well-known fact about the pivotal role of thyroid hormones in the photoperiodic response of Djungarian hamsters. We also showed that response to winter-like short photoperiod is not a binary trait, and that gene expression and tanycyte density reflected the spectrum of phenotypes. Finally, the variability observed in winter phenotypes and in neuroendocrine determinants of winter response suggests that high flexibility towards environmental fluctuations is favoured by natural selection. Since the global climate change has the potential to alter many aspects of the seasonal response, polymorphism of winter phenotype is considered to become more frequent [12]. Therefore, studies to unravel the mechanism of this phenomenon are necessary and should receive more attention of specialists in the field of seasonality research.

## Supporting information

S1 Table RNA integrity number equivalents (RIN) for RNA samples obtained from hypothalamus of experimental animals. The RNA integrity was assessed by the Genomics Core Facility of the Faculty of Medicine at Ulm University via automated gel electrophoresis (Agilent 4200 TapeStation System, Agilent Technologies Deutschland GmbH, Waldbronn, Germany). Table includes also 28S to 18S ratio and RNA concentration in the sample measured in NanoDrop (NanoDrop). The RIN numbers was assessed later, therefore we measured RNA concentration once again before RIN measurements. The concentration of RNA measured before qPCR analysis and concentration of RNA measured before RIN analysis was 0.99. See S1 Fig. Therefore we presented the first measured RNA concentration in this table.
(DOCX)

S1 Fig Correlation between concentration of RNA measured before qPCR analysis and concentration of RNA measured before RIN analysis. (TIF)

S2 Fig Effect of winter phenotype on photoresponsive index (PI; Panel A) and relative gene expression of iodothyronine deiodinase 2 (*dio2*; Panel B), iodothyronine deiodinase 3 (*dio3*; Panel C), monocarboxylate transporter 8 (*mct8*; Panel D), thyrotropin-releasing hormone (*trh*; Panel E), and somatostatin (*srif*; Panel E) in Djungarian hamsters. LP – long-photoperiod acclimated individuals, NR – non-responding individuals, PR – partial-responding individuals, FR – full-responding individuals. Numbers in brackets indicate sample size. *P <0.05.
(TIF)
