# Supplementary material for: Seasonal changes in activity of hypothalamic thyroid hormone system in different winter phenotypes of Djungarian hamster (Phodopus sungorus)
Source: PLoS One. 2024 Oct 25;19(10):e0309591. doi: 10.1371/journal.pone.0309591 (PMC11508246; doi:10.1371/journal.pone.0309591)
Supplement: S1 Table — The RNA integrity was assessed by the Genomics Core Facility of the Faculty of Medicine at Ulm University via automated gel electrophoresis (Agilent 4200 TapeStation System, Agilent Technologies Deutschland GmbH, Waldbronn, Germany). Table includes also 28S to 18S ratio and RNA concentration in the sample measured in NanoDrop (NanoDrop). The RIN numbers was assessed later, therefore we measured RNA concentration once again before RIN measurements. The concentration of RNA measured before qPCR analysis and concentration of RNA measured before RIN analysis was 0.99. See S1 Fig. Therefore we presented the first measured RNA concentration in this table. (DOCX) [file pone.0309591.s001.docx]

| **ID** | **RIN** | **28S/18S (Area)** | **RNA Conc. [ng/µl]** |
| --- | --- | --- | --- |
| 2 | 8.8 | 2.2 | 608 |
| 3 | 8.7 | 1.7 | 1016 |
| 4 | 8.4 | 2.2 | 792 |
| 5 | 8.3 | 2 | 821 |
| 6 | 8.5 | 2.2 | 693 |
| 7 | 8.5 | 2.7 | 1053 |
| 8 | 8.5 | 2.2 | 801 |
| 28 | 8.3 | 2.1 | 797 |
| 31 | 8.7 | 1.9 | 923 |
| 33 | 8.5 | 2.5 | 1083 |
| 34 | 8.4 | 2.3 | 1083 |
| 35 | 8.4 | 2.5 | 768 |
| 36 | 8.5 | 2.7 | 807 |
| 37 | 8.9 | 1.8 | 923 |
| 38 | 8.6 | 2 | 835 |
| 39 | 8.9 | 1.8 | 910 |
| 40 | 8.6 | 2.2 | 793 |
| 41 | 8.5 | 1.2 | 716 |
| 42 | 8.8 | 2.5 | 875 |
| 43 | 8.4 | 1.7 | 716 |
| 44 | 8.6 | 1.7 | 900 |
| 45 | 8.8 | 1.8 | 900 |
| 46 | 8.4 | 2.2 | 799 |
| 47 | 8.7 | 2.4 | 573 |
| 48 | 8.6 | 2.1 | 893 |
| 49 | 8.5 | 2.4 | 758 |
| 50 | 8.3 | 2.5 | 828 |
| 52 | 8.6 | 2.8 | 790 |
| 54 | 8.7 | 2.3 | 601 |
| 55K | 8.8 | 1.9 | 683 |
| 63K | 8.7 | 1.8 | 595 |
| 64K | 8.6 | 1.7 | 708 |
| 65K | 8.7 | 2.5 | 454 |
| 66K | 8.6 | 2.5 | 564 |
| 67K | 8.7 | 1.6 | 598 |
| 68K | 8.7 | 1.9 | 549 |
| 69K | 8.7 | 2.6 | 559 |
| 70K | 9 | 2.5 | 527 |
| 71K | 8.6 | 2 | 513 |
