# Supplementary figures and images for: Seasonal changes in activity of hypothalamic thyroid hormone system in different winter phenotypes of Djungarian hamster (Phodopus sungorus)
Source: PLoS One. 2024 Oct 25;19(10):e0309591. doi: 10.1371/journal.pone.0309591 (PMC11508246; doi:10.1371/journal.pone.0309591)

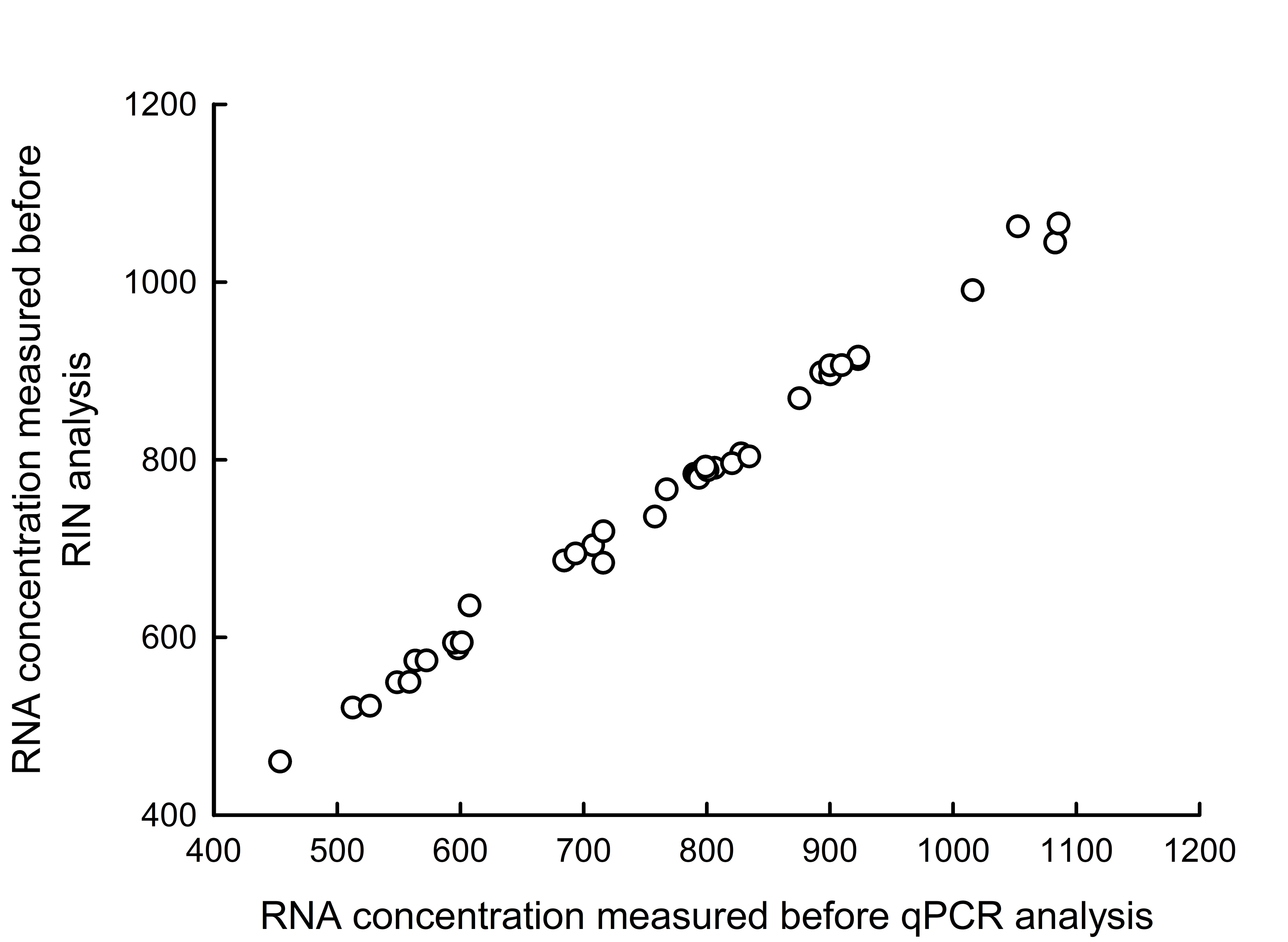

Supplement: S1 Fig — (TIF) [file pone.0309591.s002.tif]

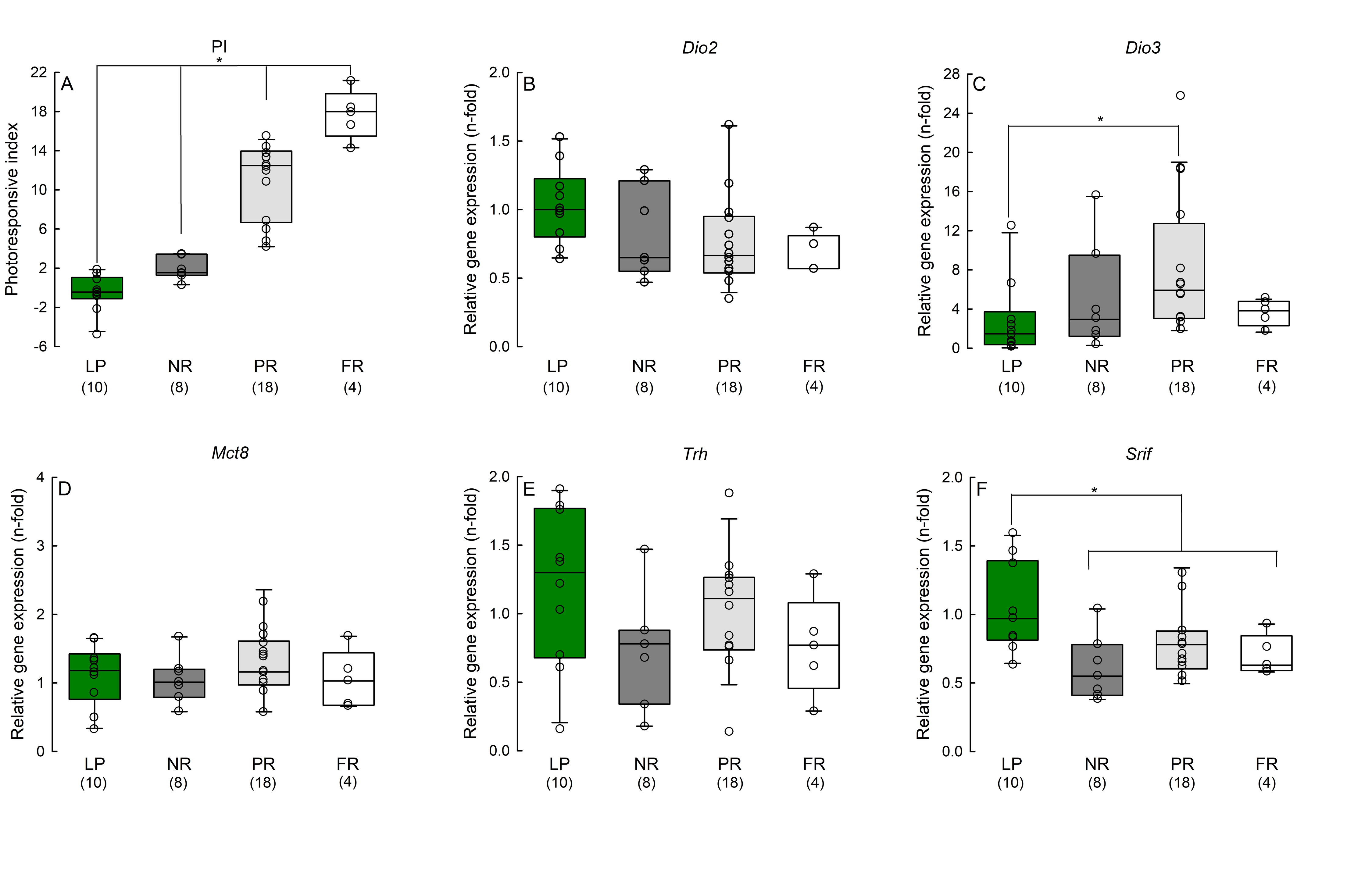

Supplement: S2 Fig — Effect of winter phenotype on photoresponsive index (PI; Panel A) and relative gene expression of iodothyronine deiodinase 2 (dio2; Panel B), iodothyronine deiodinase 3 (dio3; Panel C), monocarboxylate transporter 8 (mct8; Panel D), thyrotropin-releasing hormone (trh; Panel E), and somatostatin (srif; Panel E) in Djungarian hamsters. LP – long-photoperiod acclimated individuals, NR – non-responding individuals, PR – partial-responding individuals, FR – full-responding individuals. Numbers in brackets indicate sample size. *P <0.05. (TIF) [file pone.0309591.s003.tif]
